# Impact of Amoxicillin-Clavulanate followed by Autologous Fecal Microbiota Transplantation on Fecal Microbiome Structure and Metabolic Potential

**DOI:** 10.1128/mSphereDirect.00588-18

**Published:** 2018-11-21

**Authors:** Christopher Bulow, Amy Langdon, Tiffany Hink, Meghan Wallace, Kimberly A. Reske, Sanket Patel, Xiaoqing Sun, Sondra Seiler, Susan Jones, Jennie H. Kwon, Carey-Ann D. Burnham, Gautam Dantas, Erik R. Dubberke

**Affiliations:** a The Edison Family Center for Genome Sciences and Systems Biology, Washington University School of Medicine, St. Louis, Missouri, USA; b Division of Infectious Diseases, Washington University School of Medicine, St. Louis, Missouri, USA; c Department of Pathology and Immunology, Washington University School of Medicine, St. Louis, Missouri, USA; d Division of Gastroenterology, Washington University School of Medicine, St. Louis, Missouri, USA; e Department of Molecular Microbiology, Washington University School of Medicine, St. Louis, Missouri, USA; f Department of Pediatrics, Washington University School of Medicine, St. Louis, Missouri, USA; g Department of Biomedical Engineering, Washington University School of Medicine, St. Louis, Missouri, USA; Antimicrobial Development Specialists, LLC; University of Pennsylvania; Rush Medical College

**Keywords:** antimicrobial resistance, fecal microbiota transplantation, metagenomics, microbiome, multidrug resistance

## Abstract

Strategies to prevent multidrug-resistant organism (MDRO) infections are scarce, but autologous fecal microbiota transplantation (autoFMT) may limit gastrointestinal MDRO expansion. AutoFMT involves banking one’s feces during a healthy state for later use in restoring gut microbiota following perturbation. This pilot study evaluated the effect of autoFMT on gastrointestinal microbiome taxonomic composition, resistance gene content, and metabolic capacity after exposure to amoxicillin-clavulanic acid (Amox-Clav). Ten healthy participants were enrolled. All received 5 days of Amox-Clav. Half were randomized to autoFMT, derived from stool collected pre-antimicrobial exposure, by enema, and half to saline enema. Participants submitted stool samples pre- and post-Amox-Clav and enema and during a 90-day follow-up period. Shotgun metagenomic sequencing revealed taxonomic composition, resistance gene content, and metabolic capacity. Amox-Clav significantly altered gut taxonomic composition in all participants (*n* = 10, *P* < 0.01); however, only three participants exhibited major changes at the phylum level following exposure. In the cohort as a whole, beta-lactamase genes were enriched following Amox-Clav (*P* < 0.05), and predicted metabolic capacity was significantly altered (*P* < 0.01). Species composition, metabolic capacity, and beta-lactamase abundance returned to pre-antimicrobial exposure state 7 days after either autoFMT or saline enema (*P* > 0.05, compared to enrollment). Alterations to microbial metabolic capacity occurred following antimicrobial exposure even in participants without substantial taxonomic disruption, potentially creating open niches for pathogen colonization. Our findings suggest that metabolic potential is an important consideration for complete assessment of antimicrobial impact on the microbiome. AutoFMT was well tolerated and may have contributed to phylogenetic recovery. (This study has been registered at ClinicalTrials.gov under identifier NCT02046525.)

**IMPORTANCE** The spread of multidrug resistance among pathogenic organisms threatens the efficacy of antimicrobial treatment options. The human gut serves as a reservoir for many drug-resistant organisms and their resistance genes, and perturbation of the gut microbiome by antimicrobial exposure can open metabolic niches to resistant pathogens. Once established in the gut, antimicrobial-resistant bacteria can persist even after antimicrobial exposure ceases. Strategies to prevent multidrug-resistant organism (MDRO) infections are scarce, but autologous fecal microbiota transplantation (autoFMT) may limit gastrointestinal MDRO expansion. AutoFMT involves banking one’s feces during a healthy state for later use in restoring gut microbiota following perturbation. This pilot study evaluated the effect of amoxicillin-clavulanic acid (Amox-Clav) exposure and autoFMT on gastrointestinal microbiome taxonomic composition, resistance gene content, and metabolic capacity. Importantly, we found that metabolic capacity was perturbed even in cases where gross phylogeny remained unchanged and that autoFMT was safe and well tolerated.

## Abstract (precis)

The spread of multidrug resistance among pathogenic organisms threatens the efficacy of antimicrobial treatment options. The human gut serves as a reservoir for many drug-resistant organisms and their resistance genes, and perturbation of the gut microbiome by antimicrobial exposure can open metabolic niches to resistant pathogens. Once established in the gut, antimicrobial-resistant bacteria can persist even after antimicrobial exposure ceases. Strategies to prevent multidrug-resistant organism (MDRO) infections are scarce, but autologous fecal microbiota transplantation (autoFMT) may limit gastrointestinal MDRO expansion. AutoFMT involves banking one’s feces during a healthy state for later use in restoring gut microbiota following perturbation. This pilot study evaluated the effect of amoxicillin-clavulanic acid (Amox-Clav) exposure and autoFMT on gastrointestinal microbiome taxonomic composition, resistance gene content, and metabolic capacity. Importantly, we found that metabolic capacity was perturbed even in cases where gross phylogeny remained unchanged and that autoFMT was safe and well tolerated.

## INTRODUCTION

The spread of multidrug resistance among pathogenic organisms has rendered many treatment options ineffective. The World Health Organization has described the situation as the dawn of a postantimicrobial era (1). The human gut serves as a reservoir for many resistant organisms and their resistance genes, and this can lead to infection in the colonized host and transmission of resistance between commensals and pathogens (2–8). Once established in the gut, antimicrobial-resistant bacteria can persist for extended durations even in the absence of additional antimicrobial exposure (3–9). Novel therapeutic approaches are essential to limit or even reverse colonization with resistant organisms and the associated risk of infection and transmission between hosts.

Despite the growing antimicrobial resistance threat, there are no established methods to effectively reverse the effects of antimicrobial exposure on commensal or pathogenic bacteria. Several strategies have been proposed for mitigating the threat of resistance, but each carries risk. For example, using nonabsorbable broad-spectrum antimicrobials such as rifaximin has been proposed as a method of limiting systemic resistance selection (10). Using such orally ingested drugs to treat infections localized to the gastrointestinal tract may indeed limit systemic selection for antimicrobial resistance; however, this practice still leads to resistance selection in the gut and allows opportunistic pathogens to gain access in this critical body habitat (11).

A healthy fecal microbiome defends against pathogen and multidrug-resistant organism (MDRO) invasion through colonization resistance (12). Fecal microbiota transplantation (FMT) has been proposed as a method of restoring the microbiome to a healthy state after treatment with antimicrobials (13). By displacing infectious or resistant microbes, the new community can restore species diversity, antimicrobial susceptibility, and colonization resistance (13). FMT from healthy allogeneic donors (alloFMT) has been remarkably successful in treating Clostridium difficile infection (CDI) (13, 14). Some studies indicate that patients who receive alloFMT for CDI may have a reduction in MDROs in feces as well as infections due to intestinal colonizers (15–21). However, this approach has been found to inadvertently allow transmission of resistance genes from donor to recipient (22). Donor feces may also transmit pathogens or pathobionts which are being asymptomatically harbored by the allogeneic donor. Additionally, microbiota structure varies significantly between individuals, and a poor donor-recipient match may lead to dysbiosis or FMT failure (23–26). Studies to date of the effects of FMT on the microbiome have primarily relied on 16S ribotype-based analyses of bacterial taxonomic composition and diversity (27–30). While such studies have been transformative in demonstrating how well taxonomic compositions match between FMT donors and recipients over time, they are generally not designed to illuminate functional changes in the microbiome (29, 30). Accordingly, complementary approaches are required to enable a higher-resolution understanding of the impact of FMTs on the composition, dynamics, and transmission of resistance genes and metabolic capacity encoded by the microbiome.

Autologous fecal microbiota transplantation (autoFMT) is a potential method for restoring the gut to a healthy state while avoiding the risks of donor resistance genes and donor-recipient mismatch. AutoFMT involves storage of a healthy person’s fecal material for later use to restore the gut microbiota after perturbation, such as antimicrobial use. Bolstering the commensal microbiome by autoFMT following exposure to antimicrobials may be effective at combating colonization with MDROs (17). AutoFMT theoretically has a more desirable safety profile than alloFMT because the feces originated from the participant and was collected during a healthy state. The purpose of this study was to evaluate the effects of 5 days of amoxicillin-clavulanate (Amox-Clav) on microbiome taxonomic composition, resistance gene content, and predicted metabolic capacity and the effectiveness of autoFMT versus placebo in microbiome restoration.

## RESULTS

### Enrollment.

Ten healthy participants were enrolled in the study; participant characteristics are given in Table 1. Two participants experienced adverse events >30 days postenema: one patient was treated with antimicrobials for an ear infection, and the second was diagnosed with Helicobacter pylori infection and treated with antimicrobials. Neither infection was determined to be related to Amox-Clav, autoFMT, or saline enema. There was no difference between study groups in the number of bowel movements per day postenema or in bowel movement consistency postenema as measured by Bristol stool type (Mann-Whitney U, *P* > 0.05 for all).

**TABLE 1:** Demographics of study population, stratified by study group (*n* = 10)

Variable	Saline no. (%)	AutoFMT no. (%)
Age (median [range]), yr	34 (24–56)	26 (25–57)
Female	2 (40)	4 (80)
Nonwhite	0 (0)	1 (20)
BMI		
Underweight	1 (20)	0 (0)
Normal	2 (40)	1 (20)
Overweight	1 (20)	2 (40)
Obese	1 (20)	2 (40)
Preexisting medical condition^*a*^	2 (40)	3 (60)
Smoker (former or current)	0	0
Alcohol use		
Current	4 (80)	4 (80)
Former	1 (20)	0 (0)
Never	0 (0)	1 (20)
Special diet^*b*^	1 (20)	0 (0)
Diarrhea in the past yr	2 (40)	3 (60)
Constipation in the past yr	2 (40)	0 (0)

a Hysterectomy (two subjects), migraine headaches, mild hearing loss, history of sports injuries, arthritis, history of back surgery, history of ulcer, history of knee surgery, thyroid partial.

b Vegan plus fish.

### Taxonomic compositional analysis.

**FIG 1:**
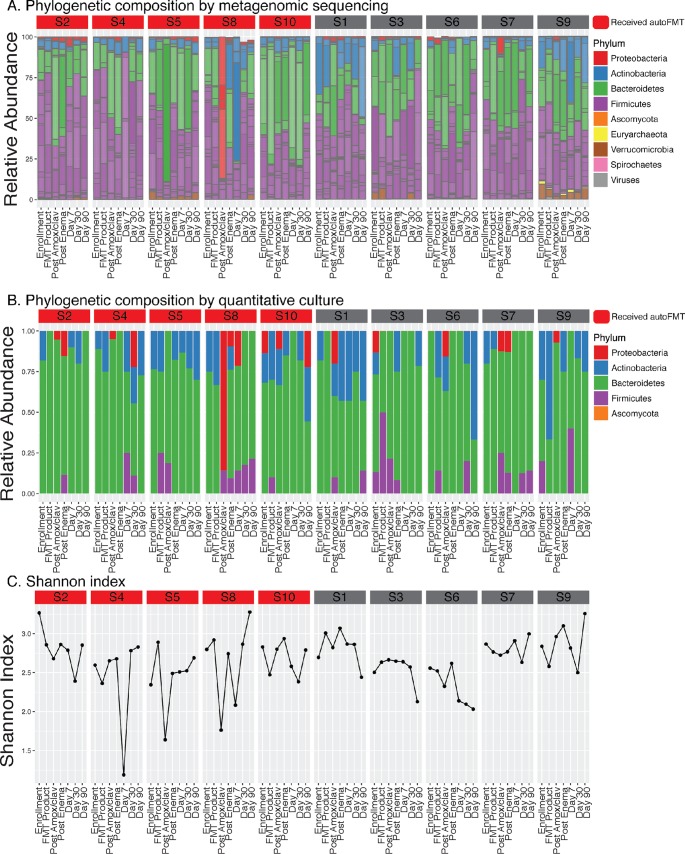
Taxonomic composition over time was determined by (A) metagenomic sequencing and (B) qualitative culturomics. (C) Shannon index of diversity was calculated using species data from metagenomic sequencing. Species composition was significantly different after Amox-Clav by type II Adonis test of Bray-Curtis distance (*P* < 0.01). The most obvious change post-Amox-Clav was a *Proteobacteria* bloom in subject 8. Numbers preceded by “S” at the top of columns indicate participant identification number.

**FIG 2:**
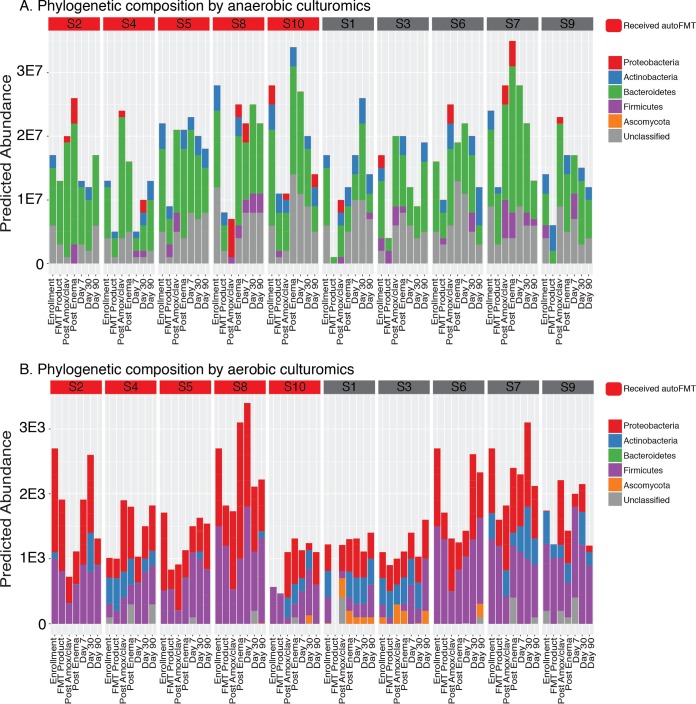
Taxonomic composition and predicted absolute abundance over time by quantitative culturomics. (A) Aerobic and (B) anaerobic cultures were quantified. Note that the aerobic cultures were less dilute, allowing greater sensitivity but a lower upper limit of detection. Also note that the relative abundance in Fig. 1B was calculated using growth observed at the greatest dilution for each species while here species may appear in both anaerobic and aerobic cultures.

Among healthy volunteers (*n* = 10), 5 days of Amox-Clav resulted in a significant taxonomic shift from the enrollment composition as measured by Bray-Curtis distance (*P* < 0.01). The taxonomic composition across all subjects and time points is visualized in Fig. 1 and 2. Both the autoFMT and saline groups returned to baseline taxonomic composition by 7 days posttreatment (Bray-Curtis distance to enrollment, *P* > 0.05).

Major taxonomic diversity reduction (Shannon index change of at least −1) occurred in three of 10 participants. Two of these shifts (in participants 5 and 8) occurred immediately following Amox-Clav. A bloom in *Bacteroidetes* and reduction in *Actinobacteria* and *Firmicutes* contributed to the diversity loss in participant 5. A proteobacterial bloom and loss of *Bacteroidetes* and *Actinobacteria* appeared to drive diversity reduction in participant 8. Both participants were randomized to the autoFMT treatment group. Additionally, participant 4 experienced a reduction in diversity due to loss of *Proteobacteria* and *Actinobacteria* 7 days after autoFMT treatment. In each of these three cases, diversity was restored by the next time point (Shannon index within 0.5 of preshift value and restoration of reduced phyla).

### Resistance gene analysis.

**FIG 3:**
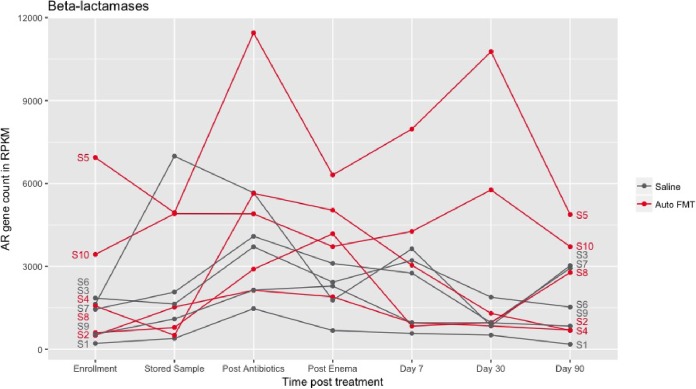
Beta-lactamase genes were significantly enriched after Amox-Clav (two-tailed *t* test, *P* = 0.0017). Participants randomized to autoFMT (red) and saline (gray) both returned to baseline by day 90. Participant numbers are noted at the beginning and end of each line.

**FIG 4:**
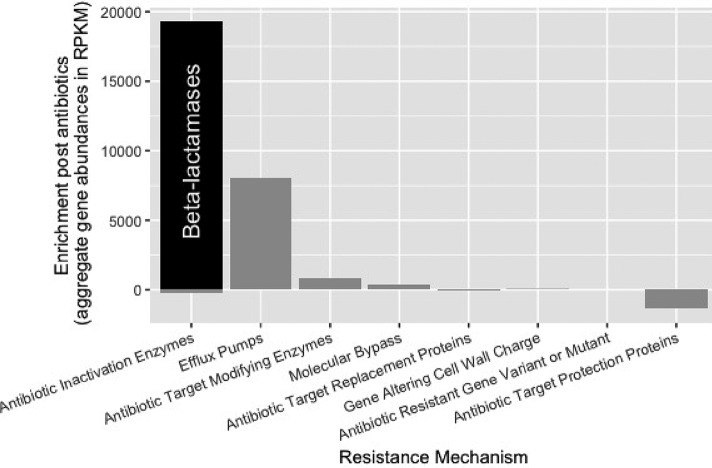
Enrichment of resistance genes by mechanism was determined by comparing normalized counts (RPKM) post-Amox-Clav to enrollment. Beta-lactamases (black) were most enriched and formed a majority of the antibiotic inactivation enzymes enriched. Efflux pumps were also enriched, and functional metagenomic selections suggest cooccurrence of beta-lactamases and efflux pumps on a mobile element.

The resistance genes detected by metagenomic sequencing included a wide range of common resistance genes. The resistance genes found across the most participants were tetracycline destructases (31, 32) and efflux pumps, but the total number of copies of resistance genes was dominated by beta-lactamases. The beta-lactamases were also the mechanistic category that showed the most change correlated with Amox-Clav treatment. As expected, the number of beta-lactamases significantly increased in the study cohort after use of Amox-Clav (*P* = 0.0017), while the count of non-beta-lactamase antimicrobial inactivation resistance genes was not enriched (Fig. 3 and 4). Beta-lactamase gene levels returned to baseline (not significantly different from enrollment level) in both the saline (*P* > 0.05) and autoFMT (*P* > 0.05) groups. The participants who experienced phylogenetic perturbation following Amox-Clav (participants 4, 5, and 8) had enriched beta-lactamase gene content following Amox-Clav treatment. Notably, these participants did not exhibit a common set of resistance genes enriched at enrollment (see Fig. S1 in the supplemental material), indicating that there is not a specific set of genes at enrollment predisposing participants to later taxonomic perturbation. The baseline beta-lactamase composition for participants 4 and 8 ranked in the middle of all enrollment samples. However, participant 5 exhibited the highest baseline beta-lactamase content.

10.1128/mSphereDirect.00588-18.1 FIG S1 Resistance gene enrichment shown by subject and time point and clustered by similarity in enrichment profile. Each column represents a unique resistance gene from the ShortBRED database or detected by functional metagenomic screening (noted by “FMG”), and each column has been normalized. Note that participants who experienced phylogenetic perturbation following Amox-Clav (subjects 4, 5, and 8) did not exhibit a common set of enriched resistance genes at enrollment. Download FIG S1, TIF file, 0.8 MB. Copyright © 2018 Bulow et al. 2018 Bulow et al. This content is distributed under the terms of the Creative Commons Attribution 4.0 International license.

Functional metagenomic selections also provided information about gene cooccurrence. Genes encoding the beta-lactamase CblA and an efflux pump from the AcrB family cooccurred on 22 different functional metagenomic contigs in eight different selections. Twelve such cooccurrences had exactly 114 bp between the genes, indicating a conserved multigene cassette.

### Metabolic capacity analysis.

**FIG 5:**
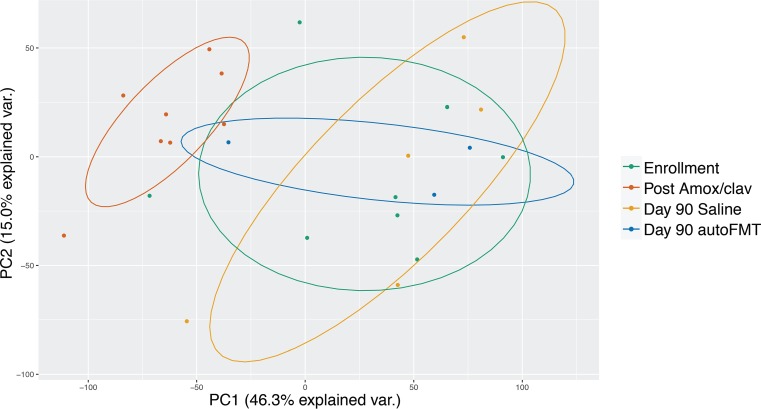
Principal coordinate analysis (PCA) of metabolic pathway data (IMC) from participants without obvious taxonomic disturbances (all participants excluding 5 and 8). IMC was derived from metagenomic sequencing data. Normally distributed confidence ellipses are shown. Post-Amox-Clav samples have significantly different IMCs than enrollment samples by type II Adonis test of Bray-Curtis distance (*P* < 0.01). This is true with or without participants 5 and 8. IMC returned to baseline state by 90 days with saline or autoFMT at similar rates.

Metabolic capacity of the microbiome shifted significantly following Amox-Clav, even when subjects with broad phylogenetic differences post-Amox-Clav (participants 5 and 8) were excluded. In order to assess metabolic perturbations, we structured an index of metabolic capacity (IMC) from the metagenomic sequencing data. Genes with metabolic functions were grouped into 37 functional categories. Principal-coordinate analysis of IMC (Fig. 5) demonstrated an effect of Amox-Clav on metabolic capacity. IMC of postantimicrobial samples clusters apart from baseline IMC. The Bray-Curtis distance between baseline and postantimicrobial IMC was significantly different by type II Adonis (*P* < 0.01). This remained true when we excluded participants with obvious taxonomic shifts at these time points (participants 5 and 8) to control for intrinsic metabolic differences between phyla (*P* < 0.01). Additionally, both saline and autoFMT groups returned to baseline composition (not significantly different from enrollment) by day seven posttreatment (*P* = 0.99 and *P* = 0.84, respectively).

Thirteen of the 37 metabolic categories comprising the IMC drove the change in metabolic capacity from enrollment to post-Amox-Clav. Each of these metabolic pathway categories was enriched post-Amox-Clav. Factors and their relative contribution are found in Fig. S2 and are ranked according to their contribution to this difference in Table S1. AutoFMT did not affect IMC differently than the saline control. IMC returned to baseline state by 30 days with saline or autoFMT. Saline or autoFMT did not result in different rates of return to baseline IMC. We found significant differences between individual participants’ IMCs when all time points were compared by type II Adonis (*P* < 0.01).

10.1128/mSphereDirect.00588-18.2 FIG S2 Metabolic features driving differences between enrollment and post-Amox-Clav samples were identified by Random Forest. Participants 5 and 8 were excluded to eliminate factors related to broad taxonomic differences. Green indicates features predicted to drive differences while red indicates features predicted to have no effect. Blue box plots indicate artificial shadow attributes used for Z score calibration (55). Download FIG S2, TIF file, 1.5 MB. Copyright © 2018 Bulow et al. 2018 Bulow et al. This content is distributed under the terms of the Creative Commons Attribution 4.0 International license.

10.1128/mSphereDirect.00588-18.3 TABLE S1 Ranked features contributing to index of metabolic capacity. Features are ranked by their predicted contribution to difference between enrollment and post-Amox-Clav IMC. Features predicted to contribute to this difference are marked “Confirmed” while features predicted to have no effect are marked “Rejected.” Download Table S1, DOCX file, 0.01 MB. Copyright © 2018 Bulow et al. 2018 Bulow et al. This content is distributed under the terms of the Creative Commons Attribution 4.0 International license.

## DISCUSSION

This pilot study evaluated the impact of Amox-Clav on the taxonomic function, resistome, and metabolic potential of the microbiome, and whether autoFMT could reverse any changes. Analysis of antimicrobial perturbation of the gut microbiome is complicated by the wide variation between baseline taxonomic compositions of healthy guts (33, 34), Additionally, while antimicrobial use has been demonstrated to reduce species diversity and cause diarrhea, the specific taxonomic changes observed vary greatly by individual (33).

Despite the dramatic and rapid perturbations observed acutely following antimicrobial exposure, surprising longer-term robustness and resilience have been previously observed. Most pilot studies and case reports after amoxicillin or ciprofloxacin indicate return to baseline by two months after exposure (33–36). However, while overall diversity and structure may be restored, some species remain missing. These analyses have been limited by resolution of 16S ribotype data and have been unable to detect resistance and metabolic genes. These factors may play critical roles in determining the robustness of the restructured microbiome to antimicrobial perturbation and pathogen invasion. Using shotgun sequencing, we tracked resistance and metabolic genes not detectable in previous 16S analyses of taxonomy.

Among healthy volunteers, 5 days of Amox-Clav exposure (875 mg BID) led to significant taxonomic shifts (*P* < 0.01), beta-lactamase gene enrichment (*P* = 0.0017), and predicted metabolic capacity alteration (*P* < 0.01). Interestingly, only two participants demonstrated obvious phylum-level taxonomic perturbation of the type readily detected by traditional analysis. However, Amox-Clav led to enrichment of 13 metabolic gene categories and significant alteration from enrollment. This significant shift in metabolic capacity was observed even in the absence of phylum-level differences. Considering that interindividual intestinal microbiome metabolic potential is more homogeneous in contrast to the interindividual phylogenic heterogeneity (37), antimicrobial-induced alterations in functional metabolic potential may be more important than taxonomic alterations when predicting acquisition and/or proliferation of an MDRO.

This study’s limitations included small sample size and limited taxonomic perturbation following Amox-Clav. In addition, the most obvious taxonomic shifts occurred in participants randomized to autoFMT. These factors limited assessment of autoFMT’s effects on taxonomic restoration. However, this provided an opportunity to assess metabolic perturbation in the absence of broad taxonomic shifts. Small sample size limited our ability to assess the implications of the observed shift in metabolic capacity. Future work is necessary to explore perturbations caused by more disruptive antimicrobial regimens and the ability of autoFMT to restore gut phylogeny.

This study assessed the effects of the dose of Amox-Clav consistent with clinical dosing and previous studies where gut dysbiosis was observed (38). This drug and dose are clinically relevant given UTI and community-acquired pneumonia treatment recommendations. Future work should assess the effects of antimicrobial protocols predicted to result in greater perturbation. In addition, methodological variations for FMT should be tested. The European Society for Clinical Microbiology and Infectious Diseases for FMT treatment of C. difficile infection recommends using 30 g of stool in 150 ml of saline (39). Although 100 ml of autoFMT product was administered in this study, 50 g of stool was used. Whether the volume administered and/or amount of stool used to create the FMT product and/or route of administration matters needs to be determined.

Despite these limitations, this study contributes to our understanding of the gut microbiome in several important ways. This study supports the concept of the healthy microbiome’s resilience following perturbation. AutoFMT was found to be safe and well tolerated, with no adverse events. By 7 days following Amox-Clav phylogeny, beta-lactamase content and predicted metabolic capacity returned to baseline.

Most strikingly, this study demonstrates the importance of assessing gut metabolic potential, which was significantly altered even in the absence of taxonomic shifts. This shift in metabolic capacity can be detected only using shotgun sequencing or metabolite profiling. Further work is necessary to determine the implications of this shift in metabolic capacity on risk of MDRO colonization and host metabolism.

## MATERIALS AND METHODS

### Overview.

This was a prospective, randomized controlled pilot study (NCT 02046525) to determine the effect of autoFMT on the intestinal microbiome versus placebo (saline enema) after 5 days of Amox-Clav, administered at 875 mg twice per day. The study participants and investigators were blinded to the treatment assignment until after all analyses were completed. This study was approved by the Washington University School of Medicine Human Research Protection Office. Written informed consent was obtained from all study participants.

### Study participants.

Ten healthy volunteers were recruited to participate. Written informed consent was obtained from all study participants. Inclusion criteria included being generally healthy and between 21 and 70 years of age. Exclusion criteria included a history of allergic reaction to beta-lactam antimicrobials or contraindications to amoxicillin-clavulanic acid; any nontopical antimicrobial exposure or tube feeds as a primary source of nutrition in the six months prior to enrollment; pregnancy or risk of becoming pregnant during the study period; gastroenteritis in the last three months; any nonelective hospitalization in the previous 12 months; incontinency of feces; prior resection or alteration of the stomach, small bowel, or colon; unwillingness to receive an enema/FMT; known colonization with an MDRO; anticipated change in diet, medications, or elective surgery during the study period; or a history of an intestinal disorder.

### Study procedures.

Participants submitted stool samples to study investigators at enrollment, immediately post-Amox-Clav, and at days 1, 7, 30, and 90 postenema. Once the preantimicrobial specimen was obtained, the participant was instructed to take 5 days of Amox-Clav at 875 mg twice daily (BID). Participants were requested to return the bottles of Amox-Clav to confirm all doses had been taken. The participant was then randomized in a 1:1 fashion to 100 ml of placebo (nonbacteriostatic saline) or autoFMT product. The autoFMT product was thawed overnight at room temperature. The study enema was administered 24 to 48 h after the last dose of Amox-Clav. The participants were instructed to retain the material for as long as possible. All participants retained >90% of the material. Study participants were monitored for 30 min after the enema. Previous studies in FMT for treatment of C. difficile infection have demonstrated that FMT effectiveness is not dependent on FMT volume (40). In order to maintain blinding of the study participant, the study enema was delivered to the study clinic in an opaque bag, and the participant was instructed to not turn around when the enema was being prepared and administered. A vial of the participant’s autoFMT product was opened in the room, regardless if randomized to placebo or autoFMT, prior to preparing the enema for administration in order to prevent the participant from attempting to guess which study group she or he had been assigned to based on the odor during the procedure. The investigators doing the sequencing work remained blinded to the study group until after the sequencing was completed.

### Fecal processing.

Participants were provided with sealable feces collection devices. After the participant collected a bowel movement, samples were delivered within 2 h of collection. Upon receipt, the feces was immediately processed with 1.5 g reserved for feces culture, 1 g for Clostridium difficile culture, and approximately 23 g for feces pulverization and sequence-based analyses, and 50 g of the subject’s first sample was used to prepare the FMT product.

### FMT product preparation.

To prepare the FMT product, approximately 50 g of feces was weighed and transferred to a sterile container. Nonbacteriostatic saline was added to the feces in a volume of twice the weight of the feces. A sterile spatula was used to emulsify the mixture for 3 to 5 min. Then the mixture was allowed to rest for 5 min. The feces-saline mixture was then poured through a stainless steel strainer to remove large particulate matter. Four 2-ml aliquots were frozen for genomic analysis of the stored FMT product. The remaining filtrate was drawn into 60-ml syringes (50 ml filtrate each). If the patient was randomized to saline, two 60 -ml syringes were filled with nonbacteriostatic saline. The filled syringes and aliquots were stored at −80°C.

### Quantitative culturomics.

Fresh feces (1.5 g) was added to an equal amount of 1× PBS and mixed thoroughly. Immediately, six 10-fold serial dilutions were made from the homogenized specimen. Ten microliters and 100 μl of the 1/10 and 1/100 dilutions were plated to two each of the following media: TSA II with 5% sheep blood (BAP; BBL BD, Franklin Lakes, NJ), Columbia colistin nalidix agar with 5% sheep blood (CNA; BBL BD), MacConkey (MAC; BBL BD), and chocolate agar (CHOC; BBL BD). The BAP and CHOC were incubated at 35°C in CO_2_. The CNA and MAC were incubated at 35°C in air. The plates were read at 24 h and 72 h. Ten microliters and 100 μl of the 1/10^3^ and 1/10^6^ dilutions were plated to two each of the following media: brucella blood agar (BBA; Anaerobe Systems, Morgan Hill, CA), *Bacteroides* bile esculin agar (BBE; Anaerobe Systems), laked blood with kanamycin and vancomycin (LKV; Anaerobe Systems), cycloserine-cefoxitin fructose agar with horse blood and taurocholate (CCFA_HT; Anaerobe Systems), and phenylethyl alcohol blood agar (PEA; Anaerobe Systems). These plates were incubated at 35°C anaerobically for 7 days. The plates were read at 48 h, 4 to 5 days, and 7 days. One gram of fresh feces was processed for culture of C. difficile, as previously described (41).

All growth was observed and recorded semiquantitatively. All distinct colonies were identified using Vitek MS IVD v2.0 MALDI-TOF MS (bioMérieux). For any isolate that was not identified, a Gram stain was performed. All isolates were stored in TSB with glycerol at −80°C.

### Metagenomic DNA extraction and sequencing.

Metagenomic DNA was extracted from 0.5 g of feces via phenol-chloroform for each sample as previously described (18). DNA from each sample was sheared to 500 to 600 bp using the Covaris E220 sonicator (intensity, 4; duty cycle, 10%; cycles per burst, 200; treatment time, 75 s; temperature, 4°C; sample volume, 130 µl). The sonication product was purified with the Qiagen PCR purification kit and eluted in 63 μl nuclease-free water (prewarmed at 50°C).

End repair and barcode ligation reactions were performed in triplicate for each sample. End repair was performed in a Bio-Rad thermocycler using the following reagents: 2.5 μl T4 DNA ligase buffer with 10 mM ATP (10×) (NEB; B0202S), 1 μl dNTP (1 mM), 0.5 μl T4 polymerase (NEB; M0203S), 0.5 μl T4 PNK (NEB; M0201S), and 0.5 μl *Taq* polymerase (NEB; M0267S).

The barcode ligation reaction was performed by adding 2.5 μl of unique sequencing barcode at 1 μM to 500 ng of end-repaired DNA. An 0.8-μl amount of T4 DNA ligase (NEB; M0202M) was incubated with the barcode and sample DNA mixture in a Bio-Rad thermocycler. Following barcode ligation, samples were pooled into groups containing 6 barcodes. Pools were purified using the Qiagen PCR purification kit and MinElute columns and eluted in 15 μl of EB.

Fragment size selection was performed using a Use 0.5 TBE 1.5% agarose gel and visualized with SYBR Safe DNA stain. Barcoded DNA fragments sized 400 to 900 bp were cut from the gel, purified with the Qiagen Gel Extraction kit and MinElute columns, and eluted in 12 μl buffer EB. Two microliters of the eluted mixture was used for PCR enrichment of size-selected products. The enrichment reaction was prepared as follows: 12.5 μl 2× Phusion HF Master Mix, 9.5 μl nuclease-free water, 1 μl Illumina PCR primer mix (F+R) (10 μM), and 2 μl gel-purified DNA. The reaction was performed in a Bio-Rad thermocycler using the following program: 17 cycles of 98°C for 30 s (0:30), 65°C for 30 s (0:30), and 72°C for 30 s (0:30); 72°C for 5 min; and 4°C forever.

Size selection of the enriched products was performed using a Use 0.5 TBE 1.5% agarose gel, and products were visualized with SYBR Safe DNA stain. Fragments sized 400 to 900 bp were cut from the gel, purified with the Qiagen Gel Extraction kit and MinElute columns, and eluted in 15 μl of EB. DNA concentration was quantified using a Qbit fluorometer, and all sample pools were combined at equal concentrations for sequencing. Prior to sequencing, pooled fragment size was assessed with a Bioanalyzer trace and barcode read distribution was assessed using a spike-in run on the Illumina sequencing platform.

To generate metagenomic sequencing reads, the Illumina NextSeq platform was used with the high output kit and settings to generate a minimum of 400 million paired-end reads per run. In total, 70 samples were sequenced at least 1 million reads per sample to allow microbiome and resistome analyses. The depth of 1 million reads was established using previous studies of the gut microbiome (42–44).

Multiplexed Illumina paired-end shotgun metagenomic sequence reads were demultiplexed by barcode. Reads without exact match to barcode were discarded. The remaining reads were quality filtered using Trimmomatic v0.35 and parameters optimized by the Dantas lab (seed mismatches, 2; palindrome clip threshold, 30; simple clip threshold, 10; minimum adapter length, 1; keep both reads, TRUE; window size, 4; required quality, 20; leading, 10; trailing, 10; minimum length, 60).

### Microbiome taxonomic composition prediction.

Taxonomic composition was determined by comparing unique indicator sequences from sequencing reads to clade-specific marker genes from approximately 17,000 reference genomes using MetaPhlAn 2.6.0 (18). Analysis was performed using metaphlan2/2.6.0 with the following parameters: –blastdb./metaphlan2/blastdb–input_type multifasta. The difference in species diversity was calculated using the Shannon index function from the R vegan package. The difference in species composition was calculated using Bray-Curtis distance between samples, and significance was tested with type II Adonis.

### Resistance gene prediction.

The metagenomic DNA sequence was analyzed for both known and sequence-novel antimicrobial resistance genes using functional metagenomic selections and curated resistance gene databases for resistance gene identification and ShortBRED for resistance gene abundance estimation (45). To supplement known resistance gene markers available via the well-curated CARD database, markers from novel, cryptic resistance gene unique to the studied samples were obtained by performing functional metagenomic selections on bacterial metagenomic DNA pooled by participant (46). Functional metagenomic identification of genes that conferred resistance to Amox-Clav was performed as previously described (18, 47, 48) by randomly shearing metagenomic DNA from each pool of feces into fragment libraries. These libraries were cloned into the natively pan-susceptible host Escherichia coli DH10B using vector PZE21. For each sample, the host cells containing library fragments were selected against amoxicillin and ampicillin at concentrations lethal to the untransformed host. Surviving colonies were pooled, and the inserted fragments were sequenced via the Illumina MiSeq platform (2 × 150 bp). Sequencing reads were assembled into contigs with the PARFUMS pipeline (49). The contigs were searched for open reading frames with MetaGeneMark (50) and annotated by hmmscan function of HMMER3 (51) against the Resfams core database (52), Pfams (46), and TIGRFAMS (46). Resulting annotations were then hand curated (46) in the following manner. Selections were excluded if >100 contigs were assembled, because this suggests a failure in the assembly or selection since <100 unique resistance contigs are expected per selection. Within each contig, annotations were ranked by specificity to the selective agents and lowest E value with preference given to Resfams annotations over Pfams or TIGRFAMS. In the absence of a clear specific causative gene annotation (i.e., a beta-lactamase), the two best annotations with <90% overlap were accepted. The sequences corresponding to accepted annotations were then pooled with known antimicrobial resistance gene sequences from CARD 2017 (46). The genes were then quantified in unassembled metagenomic sequence using the ShortBRED pipeline with a clustering identity of 1.

### Metabolic pathway prediction.

The metabolic potential of the fecal microbial communities was inferred through functional potential profiling. The presence and abundance of metabolic pathways in the microbial communities were assessed using HUMAnN2, which maps unassembled shotgun sequencing reads to functionally annotated species pangenomes in order to predict function (53). Default parameters were used, and details can be found at http://huttenhower.sph.harvard.edu/humann2.

In order to assess metabolic perturbations, we implemented an index of metabolic capacity (IMC) from the metagenomic sequencing data. To form this index, we grouped genes with metabolic functions into 37 metabolic pathway categories using Gene Ontology (GO) terms. GO term grouping was performed using ASaiM (54) and custom scripts. The ability of the predicted metabolic capacity to discriminate between sample groups was visualized using principal component analysis (PCA), and significance was tested using type II Adonis of Bray-Curtis distance between samples. This test of significance was conducted with and without participants 5 and 8 to determine whether any difference observed was driven by the large taxonomic perturbations seen in those participants. Each metabolic category comprising the IMC was tested for contribution to the difference between enrollment and post-Amox-Clav IMC using a Random Forests model. This was implemented using the Boruta package in R (55). Factors were ranked according to their contribution to this difference.

### Statistical analysis.

Significance of differences between taxonomic composition at baseline and post-Amox-Clav was calculated by determining Bray-Curtis distance between the communities and using a type II Adonis with a significance level of *P* < 0.05 with *n* = 10. Significance of differences between IMC at enrollment and post-Amox-Clav was calculated similarly. Bray-Curtis distance between IMCs was calculated, and a type II Adonis with a significance level of *P* < 0.05 with *n* = 10 was used. Because these are permutation tests, iteration cutoffs were set to detect significance up to *P* < 0.01. Saline and autoFMT treatment group taxonomy and IMC were compared to enrollment for return to baseline comparison. Enrichment of beta-lactamase genes in postantimicrobial samples relative to enrollment was calculated using a two-tailed *t* test and a significance level of *P* < 0.05. This test was also applied to the comparison between saline and autoFMT to enrollment baseline.

### Index of metabolic capacity validation.

To determine the likelihood of obtaining a significant difference in IMC between groups of microbiome samples by chance, we performed bootstrap analysis using the Human Microbiome Project 2 (HMP2) Inflammatory Bowel Disease (IBD) cohort. This publicly available data set contains 364 samples from 27 healthy participants and 375 samples from 38 ulcerative colitis patients (UC) (56). We randomly selected 2 groups of 10 healthy samples (without replacement) and saw a difference in IMC (*P* < 0.05) in 39 of 1,000 such iterations. This is consistent with the expectation that we are not likely to see a significant difference in IMC by chance alone. Note that we selected 10 samples for each group to mirror the autoFMT study design, where a significant difference in IMC (*P* < 0.01) was noted between the 10 enrollment and 10 post-Amox-Clav samples.

### Accession number(s).

All nucleotide sequences generated during this study have been uploaded to NCBI under BioProject accession no. PRJNA446061.

## References

[1] World Health Organization. 2014 Antimicrobial resistance: global report on surveillance. World Health Organization, Geneva, Switzerland.

[2] Munoz-Price LS, De La Cuesta C, Adams S, Wyckoff M, Cleary T, McCurdy SP, Huband MD, Lemmon MM, Lescoe M, Dibhajj FB, Hayden MK, Lolans K, Quinn JP 2010 Successful eradication of a monoclonal strain of Klebsiella pneumoniae during a K. pneumoniae carbapenemase-producing K. pneumoniae outbreak in a surgical intensive care unit in Miami, Florida. Infect Control Hosp Epidemiol 31:1074–1077. doi:10.1086/656243. 20738186

[3] Roghmann MC, Qaiyumi S, Schwalbe R, Morris JG Jr. 1997 Natural history of colonization with vancomycin-resistant Enterococcus faecium. Infect Control Hosp Epidemiol 18:679–680. doi:10.2307/30141505. 9350457

[4] Donskey CJ, Hoyen CK, Das SM, Helfand MS, Hecker MT 2002 Recurrence of vancomycin-resistant Enterococcus stool colonization during antibiotic therapy. Infect Control Hosp Epidemiol 23:436–440. doi:10.1086/502081. 12186208

[5] Bhalla A, Pultz NJ, Ray AJ, Hoyen CK, Eckstein EC, Donskey CJ 2003 Antianaerobic antibiotic therapy promotes overgrowth of antibiotic-resistant, gram-negative bacilli and vancomycin-resistant enterococci in the stool of colonized patients. Infect Control Hosp Epidemiol 24:644–649. doi:10.1086/502267. 14510245

[6] Zimmerman FS, Assous MV, Bdolah-Abram T, Lachish T, Yinnon AM, Wiener-Well Y 2013 Duration of carriage of carbapenem-resistant Enterobacteriaceae following hospital discharge. Am J Infect Control 41:190–194. doi:10.1016/j.ajic.2012.09.020. 23449280

[7] Schechner V, Kotlovsky T, Tarabeia J, Kazma M, Schwartz D, Navon-Venezia S, Carmeli Y 2011 Predictors of rectal carriage of carbapenem-resistant Enterobacteriaceae (CRE) among patients with known CRE carriage at their next hospital encounter. Infect Control Hosp Epidemiol 32:497–503. doi:10.1086/659762. 21515981

[8] Sethi AK, Al-Nassir WN, Nerandzic MM, Bobulsky GS, Donskey CJ 2010 Persistence of skin contamination and environmental shedding of Clostridium difficile during and after treatment of C. difficile infection. Infect Control Hosp Epidemiol 31:21–27. doi:10.1086/649016. 19929371

[9] Forslund K, Sunagawa S, Kultima JR, Mende DR, Arumugam M, Typas A, Bork P 2013 Country-specific antibiotic use practices impact the human gut resistome. Genome Res 23:1163–1169. doi:10.1101/gr.155465.113. 23568836 PMC3698509

[10] Scarpignato C 2005 Rifaximin, a poorly absorbed antibiotic: pharmacology and clinical use. Karger, Basel, Switzerland. 10.1159/000081990 15855748

[11] Rieg S, Kupper MF, de With K, Serr A, Bohnert JA, Kern WV 2015 Intestinal decolonization of Enterobacteriaceae producing extended-spectrum beta-lactamases (ESBL): a retrospective observational study in patients at risk for infection and a brief review of the literature. BMC Infect Dis 15:475. doi:10.1186/s12879-015-1225-0. 26511929 PMC4624661

[12] Vollaard EJ, Clasener HA 1994 Colonization resistance. Antimicrob Agents Chemother 38:409–414. doi:10.1128/AAC.38.3.409. 8203832 PMC284472

[13] van Nood E, Vrieze A, Nieuwdorp M, Fuentes S, Zoetendal EG, de Vos WM, Visser CE, Kuijper EJ, Bartelsman JF, Tijssen JG, Speelman P, Dijkgraaf MG, Keller JJ 2013 Duodenal infusion of donor feces for recurrent Clostridium difficile. N Engl J Med 368:407–415. doi:10.1056/NEJMoa1205037. 23323867

[14] Gough E, Shaikh H, Manges AR 2011 Systematic review of intestinal microbiota transplantation (fecal bacteriotherapy) for recurrent Clostridium difficile infection. Clin Infect Dis 53:994–1002. doi:10.1093/cid/cir632. 22002980

[15] Davido B, Dinh A, Deconinck L, de Truchis P 2017 Fecal microbiota transplantation and urinary tract infection: an interesting approach. Clin Infect Dis . doi:10.1093/cid/cix788. 29020225

[16] Crum-Cianflone NF, Sullivan E, Ballon-Landa G 2015 Fecal microbiota transplantation and successful resolution of multidrug-resistant-organism colonization. J Clin Microbiol 53:1986–1989. doi:10.1128/JCM.00820-15. 25878340 PMC4432040

[17] Buffie CG, Pamer EG 2013 Microbiota-mediated colonization resistance against intestinal pathogens. Nat Rev Immunol 13:790–801. doi:10.1038/nri3535. 24096337 PMC4194195

[18] Gupta S, Allen-Vercoe E, Petrof EO 2016 Fecal microbiota transplantation: in perspective. Therap Adv Gastroenterol 9:229–239. doi:10.1177/1756283X15607414. PMC4749851 26929784

[19] Hamilton MJ, Weingarden AR, Sadowsky MJ, Khoruts A 2012 Standardized frozen preparation for transplantation of fecal microbiota for recurrent Clostridium difficile infection. Am J Gastroenterol 107:761–767. doi:10.1038/ajg.2011.482. 22290405

[20] Hamilton MJ, Weingarden AR, Unno T, Khoruts A, Sadowsky MJ 2013 High-throughput DNA sequence analysis reveals stable engraftment of gut microbiota following transplantation of previously frozen fecal bacteria. Gut Microbes 4:125–135. doi:10.4161/gmic.23571. 23333862 PMC3595072

[21] Chang JY, Antonopoulos DA, Kalra A, Tonelli A, Khalife WT, Schmidt TM, Young VB 2008 Decreased diversity of the fecal microbiome in recurrent Clostridium difficile-associated diarrhea. J Infect Dis 197:435–438. doi:10.1086/525047. 18199029

[22] Leung V, Vincent C, Edens TJ, Miller M, Manges AR 2018 Antimicrobial resistance gene acquisition and depletion following fecal microbiota transplantation for recurrent Clostridium difficile infection. Clin Infect Dis 66:456–457. doi:10.1093/cid/cix821. 29020222 PMC5850035

[23] Bakken JS, Borody T, Brandt LJ, Brill JV, Demarco DC, Franzos MA, Kelly C, Khoruts A, Louie T, Martinelli LP, Moore TA, Russell G, Surawicz C, Fecal Microbiota Transplantation Workgroup. 2011 Treating Clostridium difficile infection with fecal microbiota transplantation. Clin Gastroenterol Hepatol 9:1044–1049. doi:10.1016/j.cgh.2011.08.014. 21871249 PMC3223289

[24] Khoruts A, Dicksved J, Jansson JK, Sadowsky MJ 2010 Changes in the composition of the human fecal microbiome after bacteriotherapy for recurrent Clostridium difficile-associated diarrhea. J Clin Gastroenterol 44:354–360. doi:10.1097/MCG.0b013e3181c87e02. 20048681

[25] Meighani A, Hart BR, Mittal C, Miller N, John A, Ramesh M 2016 Predictors of fecal transplant failure. Eur J Gastroenterol Hepatol 28:826–830. doi:10.1097/MEG.0000000000000614. 26934528

[26] Fischer M, Kao D, Mehta SR, Martin T, Dimitry J, Keshteli AH, Cook GK, Phelps E, Sipe BW, Xu H, Kelly CR 2016 Predictors of early failure after fecal microbiota transplantation for the therapy of Clostridium difficile infection: a multicenter study. Am J Gastroenterol 111:1024–1031. doi:10.1038/ajg.2016.180. 27185076

[27] Seekatz AM, Theriot CM, Rao K, Chang YM, Freeman AE, Kao JY, Young VB 2018 Restoration of short chain fatty acid and bile acid metabolism following fecal microbiota transplantation in patients with recurrent Clostridium difficile infection. Anaerobe 53:64–73. doi:10.1016/j.anaerobe.2018.04.001. PMC6185828 29654837

[28] Mintz M, Khair S, Grewal S, LaComb JF, Park J, Channer B, Rajapakse R, Bucobo JC, Buscaglia JM, Monzur F, Chawla A, Yang J, Robertson CE, Frank DN, Li E 2018 Longitudinal microbiome analysis of single donor fecal microbiota transplantation in patients with recurrent Clostridium difficile infection and/or ulcerative colitis. PLoS One 13:e0190997. doi:10.1371/journal.pone.0190997. 29385143 PMC5791968

[29] O’Toole PW, Flemer B 2017 From culture to high-throughput sequencing and beyond: a layperson’s guide to the “omics” and diagnostic potential of the microbiome. Gastroenterol Clin North Am 46:9–17. doi:10.1016/j.gtc.2016.09.003. 28164855

[30] Liu T, Yang Z, Zhang X, Han N, Yuan J, Cheng Y 2017 16S rDNA analysis of the effect of fecal microbiota transplantation on pulmonary and intestinal flora. 3 Biotech 7:370. doi:10.1007/s13205-017-0997-x. PMC5639806 29071167

[31] Park J, Gasparrini AJ, Reck MR, Symister CT, Elliott JL, Vogel JP, Wencewicz TA, Dantas G, Tolia NH 2017 Plasticity, dynamics, and inhibition of emerging tetracycline resistance enzymes. Nat Chem Biol 13:730–736. doi:10.1038/nchembio.2376. 28481346 PMC5478473

[32] Forsberg KJ, Patel S, Wencewicz TA, Dantas G 2015 The tetracycline destructases: a novel family of tetracycline-inactivating enzymes. Chem Biol 22:888–897. doi:10.1016/j.chembiol.2015.05.017. 26097034 PMC4515146

[33] Dethlefsen L, Huse S, Sogin ML, Relman DA 2008 The pervasive effects of an antibiotic on the human gut microbiota, as revealed by deep 16S rRNA sequencing. PLoS Biol 6:e280. doi:10.1371/journal.pbio.0060280. 19018661 PMC2586385

[34] Dethlefsen L, Relman DA 2011 Incomplete recovery and individualized responses of the human distal gut microbiota to repeated antibiotic perturbation. Proc Natl Acad Sci U S A 108(Suppl 1):4554–4561. doi:10.1073/pnas.1000087107. 20847294 PMC3063582

[35] De La Cochetière MF, Durand T, Lepage P, Bourreille A, Galmiche JP, Doré J 2005 Resilience of the dominant human fecal microbiota upon short-course antibiotic challenge. J Clin Microbiol 43:5588–5592. doi:10.1128/JCM.43.11.5588-5592.2005. 16272491 PMC1287787

[36] Young VB, Schmidt TM 2004 Antibiotic-associated diarrhea accompanied by large-scale alterations in the composition of the fecal microbiota. J Clin Microbiol 42:1203–1206. doi:10.1128/JCM.42.3.1203-1206.2004. 15004076 PMC356823

[37] Human Microbiome Project Consortium. 2012 Structure, function and diversity of the healthy human microbiome. Nature 486:207–214. doi:10.1038/nature11234. 22699609 PMC3564958

[38] Gillespie D, Hood K, Bayer A, Carter B, Duncan D, Espinasse A, Evans M, Nuttall J, Stanton H, Acharjya A, Allen S, Cohen D, Groves S, Francis N, Howe R, Johansen A, Mantzourani E, Thomas-Jones E, Toghill A, Wood F, Wigglesworth N, Wootton M, Butler CC 2015 Antibiotic prescribing and associated diarrhoea: a prospective cohort study of care home residents. Age Ageing 44:853–860. doi:10.1093/ageing/afv072. 26104506

[39] Cammarota G, Ianiro G, Tilg H, Rajilić-Stojanović M, Kump P, Satokari R, Sokol H, Arkkila P, Pintus C, Hart A, Segal J, Aloi M, Masucci L, Molinaro A, Scaldaferri F, Gasbarrini G, Lopez-Sanroman A, Link A, de Groot P, de Vos WM, Högenauer C, Malfertheiner P, Mattila E, Milosavljević T, Nieuwdorp M, Sanguinetti M, Simren M, Gasbarrini A, The European FMT Working Group. 2017 European consensus conference on faecal microbiota transplantation in clinical practice. Gut 66:569–580. doi:10.1136/gutjnl-2016-313017. 28087657 PMC5529972

[40] Quraishi MN, Widlak M, Bhala N, Moore D, Price M, Sharma N, Iqbal TH 2017 Systematic review with meta-analysis: the efficacy of faecal microbiota transplantation for the treatment of recurrent and refractory Clostridium difficile infection. Aliment Pharmacol Ther 46:479–493. doi:10.1111/apt.14201. 28707337

[41] Hink T, Burnham CA, Dubberke ER 2013 A systematic evaluation of methods to optimize culture-based recovery of Clostridium difficile from stool specimens. Anaerobe 19:39–43. doi:10.1016/j.anaerobe.2012.12.001. 23247066 PMC4146438

[42] Quince C, Walker AW, Simpson JT, Loman NJ, Segata N 2017 Shotgun metagenomics, from sampling to analysis. Nat Biotechnol 35:833–844. doi:10.1038/nbt.3935. 28898207

[43] Vernocchi P, Del Chierico F, Putignani L 2016 Gut microbiota profiling: metabolomics based approach to unravel compounds affecting human health. Front Microbiol 7:1144. doi:10.3389/fmicb.2016.01144. 27507964 PMC4960240

[44] Yarygin K, Tyakht A, Larin A, Kostryukova E, Kolchenko S, Bitner V, Alexeev D 2017 Abundance profiling of specific gene groups using precomputed gut metagenomes yields novel biological hypotheses. PLoS One 12:e0176154. doi:10.1371/journal.pone.0176154. 28448616 PMC5407692

[45] Kaminski J, Gibson MK, Franzosa EA, Segata N, Dantas G, Huttenhower C 2015 High-specificity targeted functional profiling in microbial communities with ShortBRED. PLoS Comput Biol 11:e1004557. doi:10.1371/journal.pcbi.1004557. 26682918 PMC4684307

[46] Jia B, Raphenya AR, Alcock B, Waglechner N, Guo P, Tsang KK, Lago BA, Dave BM, Pereira S, Sharma AN, Doshi S, Courtot M, Lo R, Williams LE, Frye JG, Elsayegh T, Sardar D, Westman EL, Pawlowski AC, Johnson TA, Brinkman FS, Wright GD, McArthur AG 2017 CARD 2017: expansion and model-centric curation of the comprehensive antibiotic resistance database. Nucleic Acids Res 45:D566–D573. doi:10.1093/nar/gkw1004. 27789705 PMC5210516

[47] Moore AM, Patel S, Forsberg KJ, Wang B, Bentley G, Razia Y, Qin X, Tarr PI, Dantas G 2013 Pediatric fecal microbiota harbor diverse and novel antibiotic resistance genes. PLoS One 8:e78822. doi:10.1371/journal.pone.0078822. 24236055 PMC3827270

[48] Pehrsson EC, Tsukayama P, Patel S, Mejia-Bautista M, Sosa-Soto G, Navarrete KM, Calderon M, Cabrera L, Hoyos-Arango W, Bertoli MT, Berg DE, Gilman RH, Dantas G 2016 Interconnected microbiomes and resistomes in low-income human habitats. Nature 533:212–216. doi:10.1038/nature17672. 27172044 PMC4869995

[49] Forsberg KJ, Reyes A, Wang B, Selleck EM, Sommer MO, Dantas G 2012 The shared antibiotic resistome of soil bacteria and human pathogens. Science 337:1107–1111. doi:10.1126/science.1220761. 22936781 PMC4070369

[50] Zhu W, Lomsadze A, Borodovsky M 2010 Ab initio gene identification in metagenomic sequences. Nucleic Acids Res 38:e132. doi:10.1093/nar/gkq275. 20403810 PMC2896542

[51] Finn RD, Clements J, Eddy SR 2011 HMMER web server: interactive sequence similarity searching. Nucleic Acids Res 39:W29–W37. doi:10.1093/nar/gkr367. 21593126 PMC3125773

[52] Gibson MK, Forsberg KJ, Dantas G 2015 Improved annotation of antibiotic resistance determinants reveals microbial resistomes cluster by ecology. ISME J 9:207–216. doi:10.1038/ismej.2014.106. 25003965 PMC4274418

[53] Abubucker S, Segata N, Goll J, Schubert AM, Izard J, Cantarel BL, Rodriguez-Mueller B, Zucker J, Thiagarajan M, Henrissat B, White O, Kelley ST, Methe B, Schloss PD, Gevers D, Mitreva M, Huttenhower C 2012 Metabolic reconstruction for metagenomic data and its application to the human microbiome. PLoS Comput Biol 8:e1002358. doi:10.1371/journal.pcbi.1002358. 22719234 PMC3374609

[54] Batut B 2016. Group abundances of UniRef50 gene families obtained with HUMAnN2 to Gene Ontology (GO) slim terms with relative abundances: release v1.2.0 (version v1.2.0). Zenodo. doi:10.5281/zenodo.50086.

[55] Kursa MB, Rudnicki WR 2010 Feature selection with the Boruta package. J Stat Softw 36. doi:10.18637/jss.v036.i11.

[56] Integrative HMP (iHMP) Research Network Consortium. 2014 The Integrative Human Microbiome Project: dynamic analysis of microbiome-host omics profiles during periods of human health and disease. Cell Host Microbe 16:276–289. doi:10.1016/j.chom.2014.08.014. 25211071 PMC5109542

