# Supplementary figures and images for: Impact of Amoxicillin-Clavulanate followed by Autologous Fecal Microbiota Transplantation on Fecal Microbiome Structure and Metabolic Potential
Source: mSphere. 2018 Nov 21;3(6):e00588-18. doi: 10.1128/mSphereDirect.00588-18 (PMC6249645; doi:10.1128/mSphereDirect.00588-18)

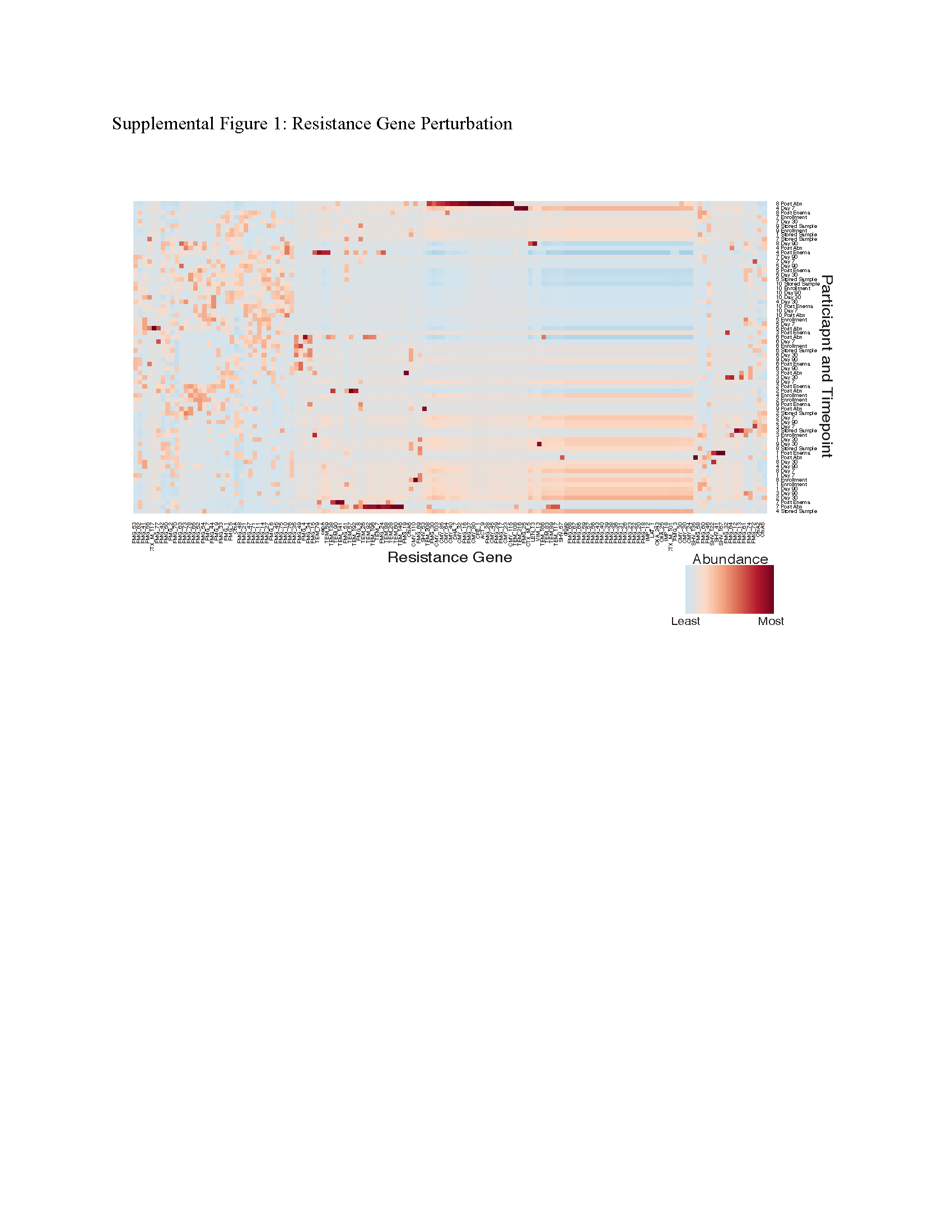

Supplement: FIG S1 [file sph006182703sf1.tif]

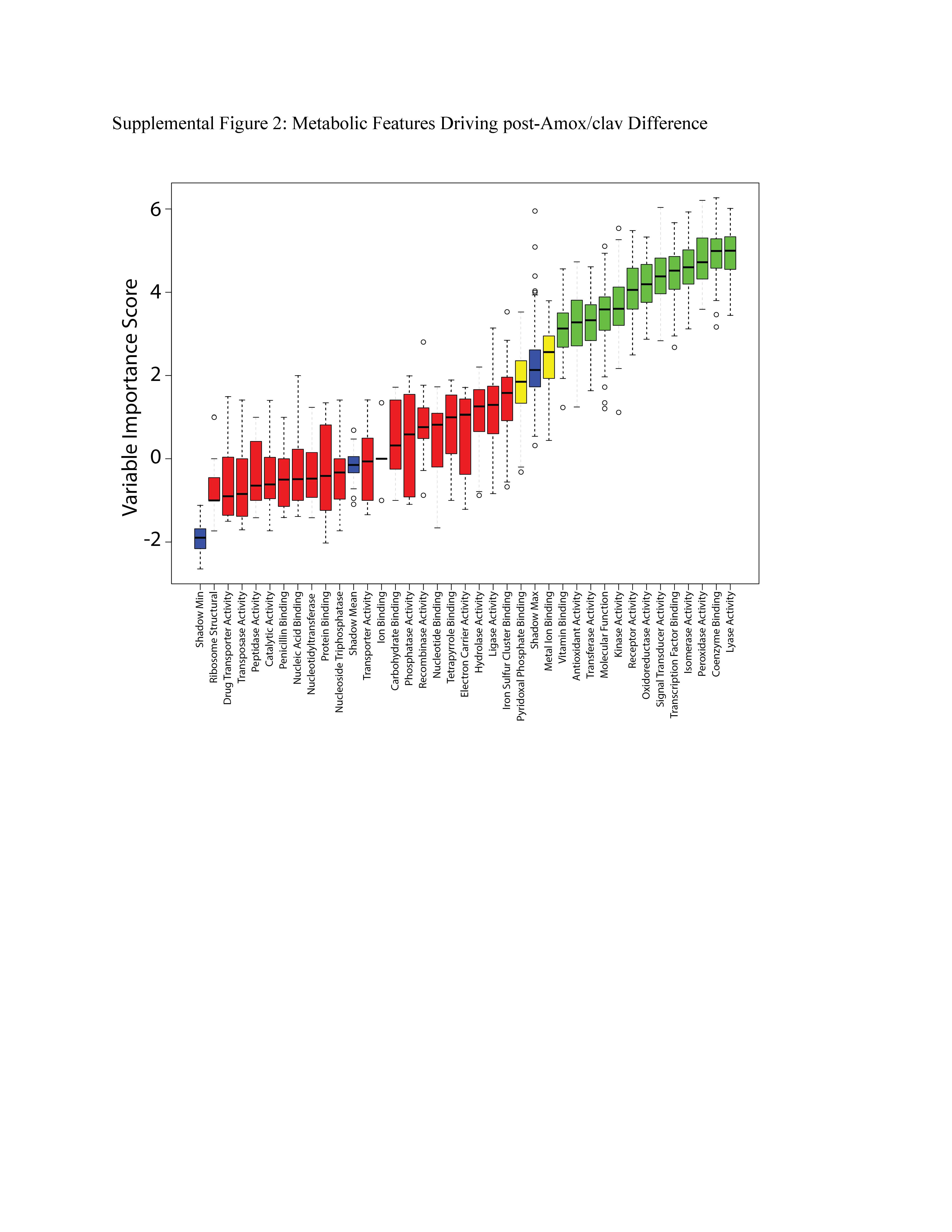

Supplement: FIG S2 [file sph006182703sf2.tif]
