# Supplementary material for: Impact of Amoxicillin-Clavulanate followed by Autologous Fecal Microbiota Transplantation on Fecal Microbiome Structure and Metabolic Potential
Source: mSphere. 2018 Nov 21;3(6):e00588-18. doi: 10.1128/mSphereDirect.00588-18 (PMC6249645; doi:10.1128/mSphereDirect.00588-18)
Supplement: TABLE S1 [file sph006182703st1.docx]

Supplemental Table 1: Ranked Features Contributing to Index of Metabolic Capacity Features

| Metabolic Category | Mean Importance Score | Decision |
| --- | --- | --- |
| lyase activity | 4.93 | Confirmed |
| coenzyme binding | 4.92 | Confirmed |
| peroxidase activity | 4.77 | Confirmed |
| isomerase activity | 4.59 | Confirmed |
| transcription factor binding | 4.44 | Confirmed |
| signal transducer activity | 4.38 | Confirmed |
| oxidoreductase activity | 4.17 | Confirmed |
| receptor activity | 4.08 | Confirmed |
| kinase activity | 3.63 | Confirmed |
| molecular_function | 3.46 | Confirmed |
| transferase activity | 3.27 | Confirmed |
| antioxidant activity | 3.23 | Confirmed |
| vitamin binding | 3.10 | Confirmed |
| metal ion binding | 2.43 | Tentative |
| pyridoxal phosphate binding | 1.81 | Tentative |
| iron sulfur cluster binding | 1.47 | Rejected |
| ligase activity | 1.24 | Rejected |
| hydrolase activity | 1.01 | Rejected |
| electron carrier activity | 0.59 | Rejected |
| tetrapyrrole binding | 0.72 | Rejected |
| nucleotide binding | 0.51 | Rejected |
| recombinase activity | 0.81 | Rejected |
| phosphatase activity | 0.45 | Rejected |
| carbohydrate binding | 0.44 | Rejected |
| ion binding | 0.03 | Rejected |
| transporter activity | -0.17 | Rejected |
| nucleoside triphosphatase activity | -0.36 | Rejected |
| protein binding | -0.29 | Rejected |
| nucleotidyltransferase activity | -0.35 | Rejected |
| nucleic acid binding | -0.28 | Rejected |
| penicillin binding | -0.42 | Rejected |
| catalytic activity | -0.44 | Rejected |
| peptidase activity | -0.37 | Rejected |
| transposase activity | -0.62 | Rejected |
| drug transporter activity | -0.60 | Rejected |
| structural constituent of ribosome | -0.74 | Rejected |
